# Meal Protein Quality Score: A Novel Tool to Evaluate Protein Quantity and Quality of Meals

**DOI:** 10.1016/j.cdnut.2024.104439

**Published:** 2024-08-17

**Authors:** Pol Grootswagers, Sine Højlund Christensen, Marielle Timmer, William Riley, Lisette de Groot, Inge Tetens

**Affiliations:** 1 Division of Human Nutrition and Health, Wageningen University, Wageningen, The Netherlands; 2 Department of Nutrition, Exercise and Sports, University of Copenhagen, Copenhagen, Denmark; 3 Wageningen Food and Biobased Research, Wageningen, The Netherlands

**Keywords:** protein quality, meal protein quality score, plant-based proteins, plant-based diets, veganism

## Abstract

**Background:**

The recent shift toward increased plant-based protein consumption has necessitated the development of new tools to evaluate the quality and quantity of protein in meals, especially given the changing dietary guidelines and the adoption of plant-centric menus in healthcare and other settings.

**Objectives:**

To develop and test the feasibility of the meal protein quality score (MPQS), a novel metric that assesses the protein quality and quantity in meals based on essential amino acid (EAA) content, digestibility, and requirements, with a focus on optimizing protein intake for vulnerable populations, particularly older adults.

**Methods:**

The MPQS integrates digestibility-adjusted EAA intake with total protein consumed in a meal, which, together with the EAA requirements, provides a score from 0 to 100 to reflect EAA coverage adequacy. The score was tested for feasibility by applying it to recipe data from real-life hospital meals and to dietary data from the [New Dietary Strategies Addressing the Specific Needs of Elderly Population for Healthy Aging in Europe] NU-AGE trial, involving detailed 7-d food records from 252 nonvegan participants analyzed over multiple meal moments.

**Results:**

The analyses revealed that the higher the content of plant protein in a meal, the lower the meal protein quality. Also, breakfast meals scored lowest on protein quality, mainly due to low contents of protein overall, and of lysine and methionine. The MPQS effectively highlighted the difference in protein quality between plant-based and animal-based meals, and across different meal types.

**Conclusions:**

The MPQS appears to be a practical tool that facilitates the assessment of meal-based protein quality. The MPQS can be used to guide dietary transitions toward plant-rich diets, ensuring that such shifts do not compromise protein adequacy for at-risk populations. The score allows for guidance in meal planning, leading to improvements in plant-rich meal formulation to meet both individual and public health nutritional needs.

## Introduction

The shift in dietary protein intake toward more plant-based proteins instead of animal-based proteins is gaining traction among consumers, employees, hospital patients, and dietary guidelines [1]. Although the beneficial effects of this transition on cardiometabolic outcomes and environmental sustainability are much welcome, it does pose a health risk to some groups of consumers [2]. These are, in general, consumers with increased protein requirements, lower food intakes, at risk of malnutrition, or at risk of sarcopenia, such as older adults and patients [3]. For these consumers, the lower anabolic properties of plant-based proteins, due to their reduced protein concentrations and quality, could increase risks of sarcopenia and osteoporosis [4].

Protein quality is a product of the digestibility, the essential amino acid (EAA) contents of a protein source, and the amino acid requirement of the individual [5]. In a diet high in animal protein, protein quality is almost never an issue [6]. Animal-based protein sources typically contain all EAAs in proportions similar to our bodily proteins and are generally >95% digestible. Hence, in typical Western dietary patterns where 60% of all proteins are consumed through animal sources [7], information on digestibility and amino acid contents was not considered crucial for human health, which explains the paucity of these data. However, official dietary guidelines are shifting to more plant-centered diets, and hospitals and meal services are more frequently offering plant-based meals to their consumers, creating a need for data and scoring algorithms to assess the protein quality of meals [8].

Calculating protein quality is not only important to plan meals and monitor the current intake but also to formulate dietary advice to improve protein quality. The recently updated Dutch recommendation for vegans to ensure adequate intake of all EAAs is to increase their protein intake by 30% above the recommendation for the general population [1], a recommendation that is not given in other Western countries. For older adults, such an increased intake is challenging, as they are already encouraged to increase their protein intake while being frequently faced with losses of appetite [3]. Also, for environmental reasons that may underlie a shift to more plant-based foods, the advice to eat more is counterintuitive.

A more elegant solution to meet EAA requirements is to combine complementary plant-based protein sources, so that they together deliver all amino acids required by the body. When meals are constructed by taking into account the EAA contents and digestibility of the meal components, a protein quality equal to that of an animal-based meal can be achieved without significantly increasing the portion size [9]. The variation in EAA profiles in plants is large, with some plant sources like peas containing significantly more lysine than the reference pattern and less methionine, whereas others like rice show the opposite trend [10]. That variation opens the possibility to mix and match sources that together deliver all EAAs that should be present in 1 meal. With data on amino acid profiles of all protein-containing foods and their digestibility, together with data on amino acid requirements and an algorithm to calculate the protein quality, many combinations of multiple plant sources that result in a high protein quality meal can be identified.

Currently, protein quality is calculated by protein digestibility-corrected amino acid score (PDCAAS) or digestible indispensable amino acid score (DIAAS). These scores take into account digestibility and amino acid patterns and were specifically developed to determine the quality of individual protein sources. However, protein sources are rarely eaten individually but normally as part of a meal with multiple protein-containing ingredients [11]. Moreover, a PDCAAS or DIAAS score does not take into account protein quantity, while having a very low intake of a high-quality source can be physiologically meaningless, as it might still be unable to meet metabolic demands. Therefore, a score is needed that reflects both the quality and the quantity of all proteins consumed together within 1 meal.

The FAO’s expert consultation on protein quality assessment recommends treating each EAA as an individual nutrient and comparing intakes to requirements when analyzing whole meals and dietary patterns [5]. That advice has previously been implemented in the EAA 9 (EAA-9) score [12]. The EAA-9 focuses on ensuring that dietary recommendations for each EAA are met. However, that score compares EAA intakes against daily requirements and is therefore suboptimal for analyzing and improving the protein quality of meals. Our proposed meal protein quality score (MPQS) addresses this gap by including meal-based EAA requirements and digestibility-adjusted amino acid intakes into a single score that is easy to interpret.

In this article, we present the development of the MPQS, which includes protein quantity, targeted EAA requirements, and digestibility-adjusted amino acid intakes in a score. We also evaluate the feasibility of using the MPQS to assess protein quality in hospital recipes and dietary intake within a large epidemiological dataset.

## Methods

### Database development

For the purpose of facilitating protein quality assessments based on Dutch dietary intake data, the food table NEVO (NEderlands VOedingsstoffenbestand 2016/5.0 [13]) was augmented to include amino acid profiles and protein digestibility data for all food items containing >1% protein. When available, ileal digestibility data were given precedence over fecal values, and data derived from human studies were prioritized over those obtained from pig and rat studies. Furthermore, digestibility data from in vitro models were not considered. The full procedure of this food table extension has been published before [14].

### Protein quality assumptions

The development of the MPQS is based on 3 key assumptions regarding protein quality, which are further examined in the discussion section:

1) Recommendation for older adults: It is recommended that older adults consume 0.3 g of high-quality protein per kilogram of body weight per meal to support muscle maintenance and overall health.
2) Protein source combinations time window: Effective protein source combinations to ensure a balanced intake of EAAs should be made within a single meal.
3) FAO/WHO reference patterns: The amino acid reference patterns provided by the FAO/WHO for >3 y old are suitable for older adults.

### Personalized EAA requirements

The MPQS takes into account personalized requirements for EAAs that a meal should deliver to optimally meet the body’s metabolic demand, which is based on a combination of a protein quantity requirement per meal and the amino acid reference patterns set by WHO [15].

For protein quantity, we use a requirement of 0.3 g/kg body weight per meal moment, based on studies that show that this amount is sufficient to stimulate muscle protein synthesis [16], and, with 3 main meals and snacks, will result in a total daily intake around 1.0–1.2 g/kg/d which is in line with official recommendations for older adults [17,18].

With this protein quantity, we have a target protein intake in grams, which we multiply by the reference amino acid patterns set by FAO/WHO [15] expressed in (mg) milligram EAA per g protein, resulting in the requirements presented in Table 1 [15].

**TABLE 1:** Essential amino acid requirements used to calculate personalized requirements.

Essential amino acid	Requirement (mg/kg bw)^1^
His	4.5
Ile	9
Leu	17.7
Lys	13.5
Met	4.8
Cys	1.8
Phe+Tyr^2^	11.4
Thr	6.9
Try	1.8
Val	11.7

Abbreviations: FAO, Food and Agriculture Organization; WHO, World Health Organization.

1 Requirements are obtained by multiplying WHO/FAO [15] essential amino acid requirements [expressed in (mg/g) milligram per gram of protein] by optimal total meal protein intake of 0.3 g/kg bodyweight.

2 Phe and Tyr are grouped together in recommendations because no individual recommendations were given by the WHO/FAO. This is due to the current inability to determine a specific value for tyrosine’s ability to spare phenylalanine intake [15].

### MPQS

Subsequently, we can score digestibility-adjusted amino acid intakes from a meal. The MPQS is a composite score that assesses protein quality and protein quantity from a meal by taking into account the amount of protein, digestibility of protein, amino acid composition, and amino acid requirement. MPQS can have values between 0 (where ≥1 EAA is completely missing in the meal) and 100 (where all EAAs reach the requirement). MPQS scores can score above 100 when each amino acid in the meal exceeds the requirement.

$$MPQS={\mathrm{MIN}\left(\%\right)}_{i}\;\left(\frac{\sum_{j}\left(intake\;\left(mg\right)\;of\;{EAA}_{i}\;from\;{food}_{j}\times digestibility\;factor\;of\;{food}_{j}\right)}{personalised\;requirement\;\left(mg\right)\;of\;{EAA}_{i}}\right)$$

As an example, Table 2 presents a meal that has an MPQS of 44, meaning that the limiting EAA meets 44% of the requirement. In this case, the digestibility-adjusted intake of methionine is 148 mg, where the meal requirement for this person of 70 kg (70∗4.8) 336 mg.

**TABLE 2:** Example calculation of meal protein quality score (MPQS). In this meal, the MPQS is 44, and the limiting essential amino acid is methionine.

	Protein digestibility, %	His	Ile	Leu	Lys	Met	Cys^1^	Phe + Tyr	Thr	Try
Ingredient A	94	204	368	628	517	102	107	653	312	103
Ingredient B	75	30	62	91	97	23	14	99	56	26
Ingredient C	64	29	58	84	92	22	6	73	63	17
TOTAL DIGESTIBILITY-ADJUSTED EAA INTAKE (mg)		263	488	803	706	148	128	825	430	146
Personalized Meal EAA Requirement (mg)^2^		315	630	1239	945	336	126	798	483	126
Requirement met by, %		83	77	65	75	**44**	102	103	89	116

Abbreviations: EAA, essential amino acid.

1 Cysteine is topped up with all methionine consumed above the requirement because methionine can be unidirectionally converted into cysteine. All amino acid intakes are adjusted for the shown digestibility factor before they are summed.

2 In this example, a body weight of 70 kg is used.

### Application of the score

The practical application of the score was tested for recipes and for large epidemiological datasets. First, recipes of meals that were provided by a hospital in the region of Copenhagen, Denmark, were calculated for protein quality by applying MPQS. The selected meals were developed to contain a higher-than-conventional proportion of plant protein. The provided recipes only included information on ingredients and quantities. Therefore, the MPQS calculations did not account for the impact of specific preparation methods or cuts, which can influence protein quality [19].

Second, the functionality of the MPQS, the score was calculated for all meals consumed at baseline by the Dutch participants of the NU-AGE trial [20]. The participants (*n* = 252) of the NU-AGE trial filled out 7 d food records, resulting in available data on 5121 meals eaten on 1757 d. The NU-AGE trial was a 1-y intervention aimed at improving the diet toward a more Mediterranean diet and had a high-quality dietary assessment [20]. Trained dietitians and nutritionists reviewed food records during home visits, ensuring completeness and accuracy through discussions with participants. Nutrients were calculated by using the NEVO food composition table [13]. For NU-AGE, ethical approval was provided by the Wageningen University Medical Ethics Committee (The Netherlands), and study procedures all complied with the ethical standards of the Helsinki Declaration. All participants gave written informed consent before participating. The trial was registered at clinicaltrials.gov (NCT01754012).

For all meals, data on MPQS, limiting amino acid (total, animal, and plant) protein intake, and energy intake were calculated. The MPQS was compared across different meal times (breakfast, lunch, and dinner) and categories of plant-based food proportions.

### Statistical methods

All presented analyses were pre-specified. Descriptive statistics are presented as means ± SD for normally distributed data and medians with (IQR; 25th percentile-75th percentile) for nonnormally distributed data. The normality of the distribution was visually inspected for all variables, and analyses were conducted using methods appropriate to each distribution type. Differences between groups were evaluated using analysis of variance followed by Tukey’s post hoc test to identify specific differences between meal moments and types. For paired comparisons, such as assessing the impact of adjustments for digestibility, the Wilcoxon signed-rank test was applied. Linear regression analysis was utilized to explore the predictive value of meal characteristics on the MPQS, whereas Spearman’s rank correlation was employed to develop a correlation matrix. All statistical analyses were conducted using SAS software, version 9.4 (SAS Institute Inc.), and GraphPad Prism, version 9.3.1 (GraphPad Software).

## Results

### Applying MPQS to assess the protein quality of recipes

A total of 22 real-life hospital meals were analyzed (Table 3). The meals were vegetarian or traditional and had proportions of plant protein varying from 15% to 69% of total protein. In meals with plant protein portions below 50%, either leucine or no EAA was limiting. In meals with greater proportions of plant protein, lysine, and methionine were limiting.

**TABLE 3:** Meal protein quality score of 22 real-world hospital meal recipes, sorted by plant portion proportion.

Recipe name	Portion size (g)	Plant/animal (mg AA/mg AA)^1^	% AA from plant^2^	MPQS^3^	Limiting EAA^4^
Focaccia with potato, and chanterelle lasagna	252	2908/1325	69	56	Methionine
Potatoes and oxheart cabbage	241	1602/925	63	33	Methionine
Cauliflower soup, rye bread, potato, cream cheese	221	2345/2226	51	56	Methionine
Mini Danish pastry with cream cheese	81	774/772	50	18	Lysine
Tartlets, rye bread, eggs	262	2755/2839	49	75	Leucine
Mushroom gnocchi, cod roe, rye bread, cream cheese	338	3795/4998	43	116	-
White bread, salad, prawns, walnut- mushroom pâté	253	2267/3719	38	81	Leucine
Sponge cake	144	1391/2365	37	51	Leucine
Asparagus soup, prawns, and rye bread with potatoes	212	1750/3710	32	73	Leucine
Greek yogurt, granola, scrambled egg with tofu, and brioche with rhubarb	240	1926/4559	30	89	Leucine
Tartlets, smoked chicken, and rye bread with eggs	325	2755/7770	26	137	**-**
Focaccia with potato, chanterelle lasagna, salmon	319	2908/8183	26	149	-
Cauliflower soup, fish terrine, rye bread with potatoes and cream cheese	267	2345/6575	26	120	-
Pommes Macaire, ham, and with Ingrid peas salad	287	1849/5337	26	92	Leucine
Rye bread with egg, potatoes, and salmon	197	1834/5299	26	91	Leucine
Macaroni with cheese, ham, and vegetarian meatballs	241	2002/6114	25	106	-
Lupin beans with rice, chicken, and vegetarian meatballs in curry	209	2071/6895	23	116	-
Potatoes, roasted beef, and oxheart cabbage	281	1602/5711	22	97	Leucine
Cannelloni, vegetarian Bolognese, and Bresaola	319	2240/7789	22	136	-
Mashed potatoes with broad beans, ham, and stew	250	1679/6170	21	90	Cysteine
Spinach and parmesan egg, bun with cheese, muesli	251	2090/8826	19	151	-
Leek pie, salmon, and rye bread with eggs	269	2008/11453	15	180	-

Abbreviations: AA, amino acid; EAA, essential amino acid; MPQS, meal protein quality score.

1 Total amino acids derived from plant and animal sources.

2 Percentage of total amino acids originating from plant sources.

3 The meal protein quality score indicates the lowest percentage of the meal requirement met by any essential amino acid. A score above 100 means that all essential amino acids exceed the meal requirement.

4 Essential amino acid with the lowest percentage in meeting the meal requirement.

### Applying MPQS to assess protein quality in a large dataset

Data from *n* = 252 participants were used, of which 56% were female, with a mean age of 71 ± 4 y (Table 4). The overall protein consumption was 1.0 ± 0.25 g/kg/d, with plant-based protein accounting for 40% ± 8% of the total protein intake. When comparing participants based on their MPQS scores, we identified a subset of 102 individuals who achieved the MPQS of 100 (indicating meeting all meal EAA requirements) in over half of their meals. This subgroup demonstrated similar demographic and physical activity levels to those who did not meet this criterion. Notable differences were observed in terms of body weight and dietary intake, with the group scoring lower on the MPQS, exhibiting higher body weights, and consuming less protein and energy overall.

**TABLE 4:** Baseline characteristics.

	Total sample (*n* = 252)	Median MPQS <100 (*n* = 150)	Median MPQS ≥100 (*n* = 102)^1^
Sex, % female	56	56	55
Age, y	71 ± 4	71 ± 4	71 ± 4
Height, cm	169 ± 8	170 ± 8	168 ± 8
Body mass, kg	75 ± 13	77 ± 15	71 ± 10
BMI, kg/m^2^	26.0 ± 3.6	26.8 ± 4.0	25.0 ± 2.7
PASE score	137 ± 53	136 ± 54	136 ± 54
Protein intake, g/d	76 ± 16	69 ± 13	86 ± 15
Protein intake, g/kg/d	1.0 ± 0.2	0.9 ± 0.2	1.2 ± 0.2
Of which plant protein, %	40 ± 8	41 ± 9	39 ± 8
Energy intake, kcal/d	1908 ± 411	1800 ± 366	2070 ± 422
Energy intake, mJ/d	8.0 ± 1.7	7.5 ± 1.5	8.7 ± 1.8
Percentage of meals >100 MPQS	44 ± 18	33 ± 11	61 ± 12
Median MPQS score	91 [71–112]	74 [61–87]	119 [107–134]

Abbreviations: BMI, body mass index, MPQS, meal protein quality score; PASE, physical activity scale for the elderly. ^1^A median MPQS of ≥100 indicates that more than half of the meals consumed by an individual meet all essential amino acid requirements.

### Inspection of missed proteins

A mean of 11.1 ± 10.9 g protein was consumed outside of the 3 main meals and thus not considered in the calculation of median daily MPQS. Out of the 1757 d that were analyzed, on 263 d (15%), the amount of protein eaten outside of the main meals exceeded 20 g. Inspection of this 263 d revealed that the proteins mainly come from small snacks (cake, nuts, cheese, etc.) and from milk. Only on 10 d (<1%) breakfast was consumed at the meal moment “before breakfast” and thus missed in the analyses.

### MPQS per main meal moment

**FIGURE 1:**
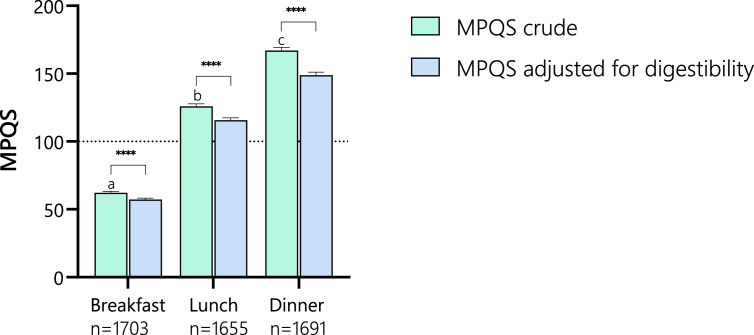
Meal protein quality score (MPQS) by meal moment. Note that the MPQS is significantly lower when adjusted for protein digestibility. Additionally, there is a clear pattern of increasing MPQS across the main meal times throughout the day. The dashed line at MPQS 100 marks the threshold where all essential amino acids meet the meal requirement. ∗∗∗∗ *P*-value <0.0001.

Figure 1 shows the mean MPQS scores per main meal moment before and after adjustment for digestibility. Clearly, MPQS increases over the meal moments, with the lowest score (at breakfast (57 ± 1) and the highest at dinner (149 ± 2). Digestibility adjustment significantly lowered the MPQS in all meal moments [median decrease in MPQS of 4 (6.5%), 8 (6.3%), and 17 (10.1%) for breakfast, lunch, and dinner, respectively]. The most frequent limiting EAAs at breakfast were lysine (73%) and methionine (18%, Table 5).

**TABLE 5:** Frequency percentage of limiting essential amino acid per meal moment and plant protein category.

	Breakfast	Lunch	Dinner	Plant protein 0%–27%	Plant protein 27%–65%	Plant protein 65%–99%	Plant protein 100%
Cysteine	8	11	18	42	8	1	9
Leucine	2	10	27	22	10	2	1
Lysine	73	61	36	4	60	89	80
Methionine	17	16	18	31	21	7	11
Threonine	-	<1	-	-	<1	-	-
Valine	<1	1	<1	1	1	-	-

### MPQS per plant protein proportion

**FIGURE 2:**
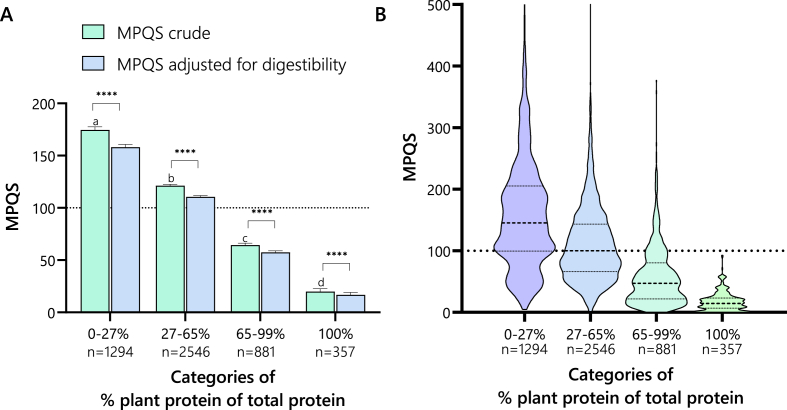
(A) Mean meal protein quality score (MPQS) by plant protein proportion. This figure illustrates that meals composed entirely of plant-based proteins tend to have lower protein quality scores. Notably, within the group of meals with 100% plant protein, breakfasts constitute 71% and are generally associated with lower protein quality scores. This differentiation highlights the influence of meal type on protein quality assessments in plant-based diets. (B) MPQS distributions by plant protein proportion. This figure presents a violin plot illustrating the distribution of MPQSs across different proportions of plant protein. It highlights that meals exclusively comprising plant proteins do not achieve a score of 100, whereas meals with a high proportion of animal proteins frequently exceed a score of 100, suggesting instances of protein overconsumption. ∗∗∗∗ indicates *P* <0.0001 and the dashed line indicates MPQS of 100.

Figure 2 shows the mean Figure 2A and distribution Figure 2B of MPQS per plant protein proportion. MPQS decreases with increasing proportions of plant-based protein of total protein in a meal. From all *n* = 357 fully vegan meals, no meal reached an MPQS of 100, indicating that all these meals are inadequate in some EAA. The limiting EAAs of the vegan meals were [expressed as (%) a percentage of cases] lysine (79%), methionine (11%), cysteine (9%), and leucine (1%, Table 5). Inspection of these vegan meals showed that 71% of them were breakfast meals. The MPQS of all animal-rich meals frequently scored above 100, with extremes reaching 500, meaning that the consumption of the limiting EAA exceeds the requirement by 5 times.

**FIGURE 3:**
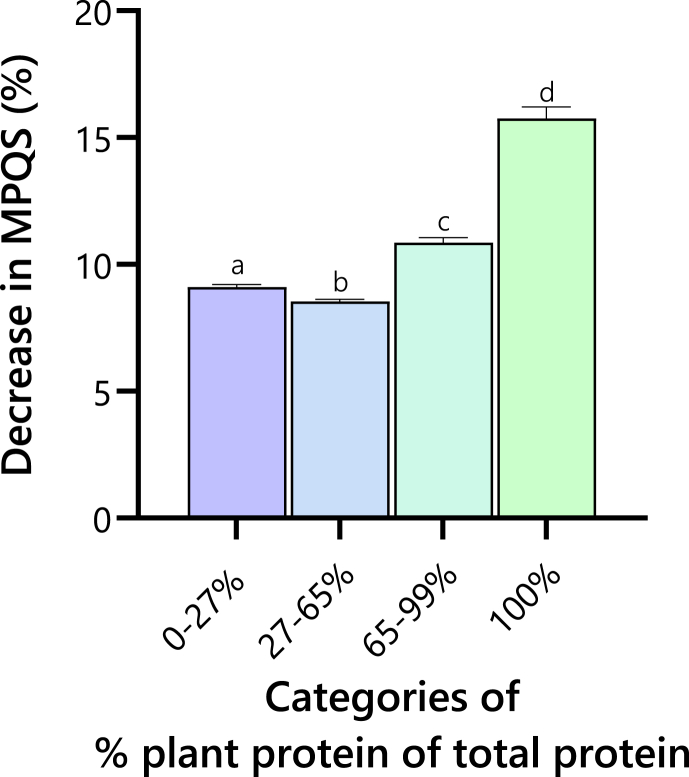
Impact of digestibility adjustment on the meal protein quality score (MPQS) of meals by plant protein proportion. This figure demonstrates how the inclusion of a digestibility adjustment factor affects the MPQSs, particularly as the proportion of plant protein in meals increases. The relevance of digestibility adjustment is accentuated in meals with higher plant protein content, illustrating the significant role that digestibility plays in evaluating the protein quality of plant-based meals.

Digestibility adjustment significantly reduced MPQS in all categories of plant protein proportion. Figure 3 shows that the impact of digestibility adjustment is around 10% in the meals containing animal protein but above 15% in the vegan meals.

### Correlation of meal nutrients and MPQS

**FIGURE 4:**
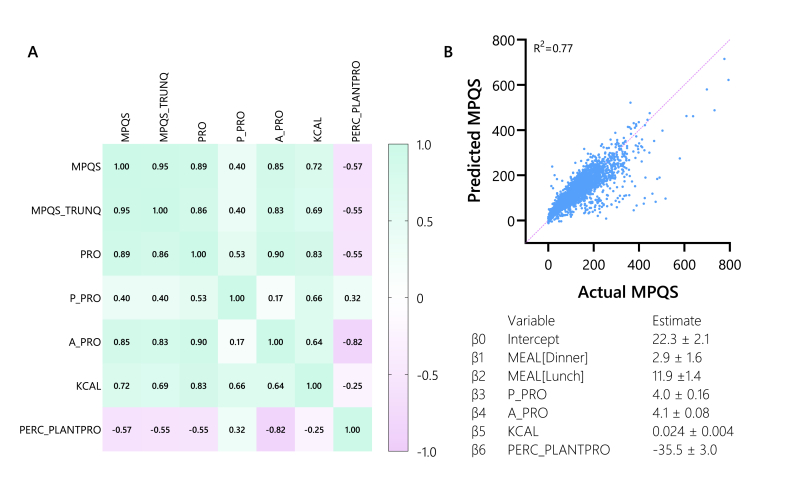
(A) Spearman’s correlation matrix for meal protein quality score (MPQS) and nutritional characteristics. (B) Predicted compared with actual MPQS. This figure illustrates that the MPQS can be accurately predicted using a combination of factors, including meal timing (compared with breakfast), the quantity of plant and animal proteins, total kilocalories, and the percentage of plant protein. A_PRO, animal protein in gram; KCAL, kilocalories; MPQS, meal protein quality score; MPQS_TRUNQ, meal protein quality score truncated at 100; P_PRO, plant protein in gram; PERC_PLANTPRO, percentage of total proteins in meal originating from plant sources; PRO, protein in gram.

Figure 4A shows the correlation matrix between MPQS and several meal nutrient characteristics. MPQS was shown to be positively correlated with amounts of total protein, plant protein, animal protein, and kilocalories in a meal, whereas the percentage of meal plant protein content showed a negative correlation. Linear regression showed that a model containing meal moment information and nutritional composition information was able to predict MPQS with an R^2^ of 0.77 (Figure 4B).

## Discussion

This article describes the development of a new protein quality score that combines amino acid patterns, protein digestibility, timing of combinations, and protein quantity. We show how the score can be applied to make the protein quality of meals insightful and use it as a starting point for meal improvement to ensure sufficient protein quality. Additionally, we show how it can be used to quantify the protein quality of individual dietary intake in large nutritional datasets and in real-world recipes.

Several protein quality scores already exist, such as PDCAAS and DIAAS. These scores are suitable for protein quality assessment at the product level only, whereas, in practice, people eat meals in which specific combinations of protein products are made [11]. Other scores have been developed to address this limitation of PDCAAS and DIAAS, such as the EAA-9 score [12], which enables protein quality calculations similar to our developed scoring algorithm. The EAA-9 scoring framework, however, does not strictly account for the timeframe within which protein combinations must occur, nor does it establish clear amino acid requirements per specific time intervals. As a result, although the EAA-9 remains highly flexible, the numerous decisions required of the user may impede practical implementation.

Our score relies on several assumptions. First, it assumes a meal protein recommendation of 0.3 g of perfect quality protein per kilogram body weight per meal. That number is based on several arguments. First, it seems that this protein amount per meal is sufficient to stimulate muscle protein synthesis [16]. There are studies suggesting that an even higher dose of proteins would be ideal for older consumers [21], but we reason that we should err on the lower bound of the ideal protein range. That is because we optimize the protein quality within this quantity goal, meaning that we may achieve higher levels of muscle protein synthesis at lower amounts of total protein quantity. Moreover, it seems inappropriate to try and find meal protein doses where muscle protein synthesis is stimulated *optimally* or *maximally* [22], as the latest evidence seems to point out that such levels could very well exceed 100 g of protein per meal [23]. Finally, with 3 meals of 0.3 g of optimized protein per kilogram bodyweight and some protein intake coming from snacks, we will achieve intakes of ≥1 g of optimized protein per kg bodyweight. There is no indisputable evidence that a daily protein intake well above 1 g/kg of body weight provides health benefits for older individuals [24]. Although moderately increased intakes do not appear to pose health risks, it is imprudent to recommend unnecessarily high intakes due to the environmental impact of protein production.

A second assumption is that combinations of protein sources should be made within 1 meal. Current research does not clearly point to a specific time frame in which protein sources must be combined to complement each other’s amino acid profiles [1]. Some experts argue that this combination should ideally occur within a meal, especially on lower protein diets or when specific EAAs are limited on sequential days [25]. Others believe protein combinations can be spread over an entire day [26]. Although there are no strict storage pools for free amino acids, recent work by Pinckaers et al. [27,28] suggests that the body may correct for an unbalanced amino acid composition when consuming large doses of protein. Their results indicate that missing amino acids may be compensated for by plasma pools, although the precise mechanism remains insufficiently elucidated. In pigs, labile storage proteins have been proposed as a potential mechanism supporting this amino acid buffering feature [29,30]. However, the capacity to correct an amino acid imbalance was limited, as the total amount of EAAs appearing in the plasma of the portal vein was still greater when comparing whey to gelatin [30]. Consequently, excessive amino acid imbalances likely result in suboptimal utilization of ingested proteins, and labile storage proteins have not yet been identified in humans.

Interestingly, a recent study explored the impact of daily supplementation with 50 g of protein from whey, pea, and collagen on muscle protein synthesis in older adults over a week [31]. The findings revealed that, unlike whey and pea proteins, collagen protein did not enhance muscle protein synthesis. The authors suspect that the low leucine content in collagen protein may be responsible for its inability to promote muscle protein synthesis. However, it is also plausible that the complete absence of the EAA tryptophan in collagen (resulting in an MPQS of 0) contributes to its failure to stimulate muscle protein synthesis, thereby supporting the notion that a meal must contain all EAAs in the proper balance to effectively stimulate protein synthesis. Until the debate around the time window of protein complementation is settled, we err on the strict side and assume the meal moment is the time window in which protein sources should be combined. If needed, our scoring mechanism can be adapted into a daily protein quality score by inputting cummulative daily amino acid intake and using recommended daily allowances (RDAs) or a combination of a daily protein target (such as 0.8 or 1.2 g/kg/d) with an amino acid reference pattern.

Thirdly, we assume that the amino acid reference patterns recommended by FAO and WHO represent ideal quantities for older adults. These reference patterns are established for the total population, whereas older adults may benefit from higher amounts of certain amino acids, such as leucine [32]. Moreover, where other scoring algorithms combine methionine and cysteine by simply using their sum, we use a more complex assumption where only methionine consumed above the recommendation will be converted into cysteine and never the other way around [33]. That is justified based on metabolic possibilities. However, some studies suggest that the methyl-donating capacities of the sulfuric amino acids are the main driver of their physiologic role, suggesting that using their sum could be appropriate after all [34].

In this study, we observed that more plant-based meals are often of lower quality. That is in line with many previous observations. Importantly, this study did not specifically include vegetarians and vegans, who may be experienced in making better combinations and thus achieve higher protein quality in their predominantly plant-based meals. Moreso, our study sample tended to consume the majority of their animal proteins at dinner, and the lowest at breakfast. In our observations, over 70% of the vegan meals were breakfasts. Breakfasts are known to be the lowest-scoring meals in terms of protein quantity [35] and quality, therefore confounding the relationship between the plant protein proportion of a meal and its protein quality.

Moreover, dividing the study population in median MPQS above or below 100 revealed that the high MPQS group had a 15% higher energy intake and a 25% higher protein intake, suggesting generally higher consumption levels, particularly of protein. Interestingly, the BMI (in kg/m^2^) in the high MPQS group was 7% lower compared to the low MPQS group. This disparity suggests potential underreporting of energy intake, yet it does not account for the notably higher protein consumption observed in the high MPQS group. Possibly, a higher protein quality intake has an effect on body composition by a larger stimulation of fat free mass synthesis, but that hypothesis needs to be investigated.

In future work, we propose to validate the MPQS by correlating meal intakes of varying quality scores with longitudinal changes in muscle mass or bone mineral density. Alternatively, direct validation can be conducted through muscle protein synthesis assessment using stable isotope techniques after consuming meals of low, medium, and high quality to determine dose-response relationships. Additionally, we aim to improve our protein digestion corrections by shifting from fecal digestion assessments to ileal digestion corrections for each EAA.

In conclusion, we present a new scoring algorithm that enables the calculation of protein quality of meals and recipes: the MPQS. Our new score is of added value in the field of protein quality, where it can guide the protein transition toward plant-rich meals of high quality so that vulnerable populations such as older adults and patients can safely transit to more plant-based diets as well. We show that the MPQS can be used to qualify and improve recipes, calculate individual protein quality intake per meal, and calculate the protein quality of all meals in large epidemiological studies.

## Author contributions

The authors’ responsibilities were as follows – PG, SHC, MT, WR, LdG, IT: designed research; PG: conducted research, analyzed data, and wrote the article; SHC, MT, WR, IT, LdG provided essential materials; and all authors: read and approved the final manuscript.

## Conflict of interest

The authors report no conflicts of interest.

## Data Availability

Data described in the manuscript, code book, and analytic code will be made available upon request, pending application and approval.
